# Negative affectivity and social inhibition are associated with increased cardiac readmission in patients with heart failure: A preliminary observation study

**DOI:** 10.1371/journal.pone.0215726

**Published:** 2019-04-19

**Authors:** Tin-Kwang Lin, Kai-Xun You, Chiu-Tien Hsu, Yi-Da Li, Chin-Lon Lin, Chia-Ying Weng, Malcolm Koo

**Affiliations:** 1 Division of Cardiology, Department of Internal Medicine, Dalin Tzu Chi Hospital, Buddhist Tzu Chi Medical Foundation, Dalin, Chiayi, Taiwan; 2 School of Medicine, Tzu Chi University, Hualien City, Hualien, Taiwan; 3 Department of Psychology, National Cheng Chung University, Minxiong, Chiayi, Taiwan; 4 Clinical Psychology Center, Dalin Tzu Chi Hospital, Buddhist Tzu Chi Medical Foundation, Dalin, Chiayi, Taiwan; 5 Department of Adult and Continuing Education, National Chung Cheng University, Minxiong, Chiayi, Taiwan; 6 Graduate Institute of Long-term Care, Tzu Chi University of Science and Technology, Hualien City, Hualien, Taiwan; 7 Dalla Lana School of Public Health, University of Toronto, Ontario, Canada; Universita degli Studi di Napoli Federico II, ITALY

## Abstract

**Background:**

Type D personality was hypothesized to influence clinical and patient-centered outcomes patients with heart failure. The aim of this study was to investigate the association between negative affectivity and social inhibition components of Type D personality and cardiac readmission in patients with heart failure.

**Methods:**

A prospective observational study design was used. A total of 222 patients with heart failure were recruited from the department of cardiology in two regional hospitals in Taiwan. The 14-item Type D Scale-Taiwanese version was used to assess negative affectivity and social inhibition of the patients. Logistic regression analyses were conducted to determine the association of both Z-score transformed and dichotomized negative affectivity and social inhibition with 6-month and 18-month cardiac readmissions.

**Results:**

A total of 55 patients (24.8%) and 89 patients (40.1%) had cardiac readmissions within 6 months and 18 months, respectively. Multiple logistic regression analyses of Z-score transformed negative affectivity and social inhibition were significantly associated with (1) 6-month cardiac readmission with odds ratios of 1.62 (P = 0.003) and 1.48 (P = 0.014), respectively and (2) 18-month cardiac readmission with odds ratios of 1.45 (P = 0.013) and 1.38 (P = 0.031), respectively. Similar findings were obtained when negative affectivity and social inhibition were analyzed as dichotomized scores.

**Conclusions:**

Negative affectivity and social inhibition components of the Type D personality were significantly associated with a higher risk of cardiac readmission in both 6 months and 18 months after the initial hospitalization in patients with heart failure.

## Introduction

Heart failure is chronic, progressive condition in which the heart is unable to provide adequate blood flow to meet the body’s needs for blood and oxygen. It is a pandemic affecting at least 26 million people worldwide [1]. The prevalence of heart failure was estimated to increase from 0.7% in persons aged 45–54 years to over 10% in those aged 85 years or older [2]. In the United States, an estimated 5.7 million people have heart failure, and the annual cost for treatment of heart failure has been estimated to be over 32 billion US dollars [3]. In addition, heart failure is a common cause of unplanned hospital admissions and readmissions [4]. About half of the patients with heart failure were readmitted to a hospital within 6 months of discharge [5]. Unplanned readmissions after initial hospitalization can significantly increase the costs of health care and affect the quality of life in patients with heart failure [6, 7]. Therefore, there is an ongoing need to identify factors to reduce readmissions after heart failure hospitalization [8].

A systematic review examined patient-level characteristics associated with readmission and found that age and sex were the most commonly included variables in readmission models, but there was no consistent association among other covariates, such as diabetes mellitus and hypertension. An increased in the biomarker B-type natriuretic peptide was shown to be consistently associated with readmission [9]. Another systematic review revealed that noncardiovascular comorbidities, poor physical condition, a history of admission, and failure to use evidence-based medication were significantly associated with 90-day readmission in patients with heart failure [10]. A study based on the secondary analysis of an administrative database in Japan showed that older age, lower body mass index, higher New York Heart Association (NYHA) functional classification at admission, higher Charlson Comorbidity Index, shorter length of stay in a hospital, and use of beta blockers, loop diuretics, thiazide, and nitrates at discharges were associated with an increased risk in 30-day readmission in patients with heart failure. Conversely, the use of angiotensin-converting enzyme inhibitors (ACEs) or angiotensin II receptor blockers (ARBs), calcium-channel blockers, and spironolactone were associated with a decreased risk [11].

Recently, it has been hypothesized that Type D personality could be associated with clinical and patient-centered outcomes and self-care in patients with heart failure, possibly through the involvement of the sympathetic nervous system, inflammation, and oxidative stress pathophysiological pathways [12]. Nevertheless, the evidence is less consistent compared with the association between Type D personality and coronary artery disease [13–15]. The distressed personality construct (Type D) is a relatively stable personality disposition that refers to the joint tendency towards negative affectivity (NA) and social inhibition (SI). Individuals with high NA scores exhibit a tendency to experience negative emotions, while those with high scores on SI tend to inhibit their emotions and behaviors in social situations [16]. While there were studies evaluating the Type D personality and medication adherence [17] and self-care behaviors [18] in patients with heart failure, to the best of our knowledge, no studies have investigated the association between Type D personality and the risk of cardiac readmission in these patients. Therefore, the aim of this study was to investigate the association between NA and SI components of Type D personality and cardiac readmission in patients with heart failure.

## Methods

### Study design and participants

A prospective observational study design was used to assess the factors associated with 6-month and 18-month cardiac readmission in patients with heart failure. Using convenience sampling, inpatients with heart failure were recruited from the department of cardiology in two regional teaching hospitals in Taiwan. Inclusion criteria included patients with a NYHA functional classification ≥ 2 and data on left ventricular ejection fraction (LVEF) available within 6 months of the admission. Patients with the following conditions were excluded: cancer, bedridden for over 3 months, severe visual or hearing impairment, and psychiatric disorders. Patients with psychiatric disorders were ascertained by a self-reported questionnaire with questions on whether they had received any mental health care in the past or had any physician-diagnosed psychiatric disorders. A total of 269 patients were eligible for the study but only 222 patients with included in the analysis. Forty-seven patients were excluded due to the following reasons: death (2 patients), incomplete questionnaire (21 patients), and lost to follow-up or refused to participate in the study during follow-up (24 patients).

The study protocol was approved by the Institutional Review Board of Dalin Tzu Chi Hospital, Buddhist Tzu Chi Medical Foundation, Taiwan (No. B10004009-1). Written informed consent was obtained from all participants.

### Questionnaire

The 14-item Type D Scale-Taiwanese version (DS14-T) [19], originally developed by Denollet [13] was used to assess NA and SI of the patients. The NA and SI components each contain seven items with a 5-point Likert scale (0–4 range). The original scale showed good psychometric properties (Cronbach α = 0.88 and 0.86; 3-month test-retest reliability = 0.72 and 0.82 for NA and SI, respectively) [13]. The DS14-T also demonstrated good internal consistency (Cronbach α = 0.86 and 0.79), with factor analyses confirmed the two-factor model of the Type D construct [19]. Since the issue whether Type D personality is better represented by a continuous or dichotomous construct is yet unresolved [20–22], separate analyses using either continuous scores or the dichotomous classification for NA and SI were conducted in this study.

Information on age, sex, body weight, body height, smoking habit, LVEF, NYHA functional classification, comorbidity, and medication use were also ascertained from medical records.

### Outcome measurement

Unplanned cardiac-related hospital readmission 6 months and 18 months following discharge were the main outcomes of this study. Cardiac readmission was defined based on

International Statistical Classification of Diseases and Related Health Problems, 9th revision, (ICD-9) diagnosis codes 390–398, 401–405, 410–414 and 420–429 or patients when admitted to cardiac wards (either internal medicine or surgical ward). Readmissions within 6 months or 18 months were ascertained either from the electronic medical records database of the study hospital or by telephone interview.

### Statistical analysis

Categorical variables were expressed as number with percentage and continuous variables as mean and standard deviation. To aid the interpretation of the results for NA and SI, their values were transformed Z-scores with a mean of zero and a standard deviation (SD) of 1. Univariate and multiple logistic regression analyses were conducted to determine the association of Z-score transformed NA and SI with 6-month and 18-month cardiac readmissions. In addition, NA and SI were also analyzed as dichotomized scores using the standard cut-off of ≥ 10 [13]. Independent variables evaluated during model development included: sex, age, body mass index, smoking, left ventricular ejection fraction, New York Heart Association functional classification, Beck Depression Inventory, type 2 diabetes, kidney disease, hypertension, coronary heart disease, arrhythmia, diuretics, beta-blockers, angiotensin-converting enzyme inhibitors, and angiotensin II receptor blockers. Backward elimination method based on likelihood ratio test was used for selection of covariates in the multiple logistic regression models. All analyses were performed with SPSS (version 24.0) (SPSS Inc., Chicago, IL, USA), and a *P*-value of 0.05 was considered significant.

## Results

The basic characteristics of the study participants at baseline are presented in Table 1. Patients with Type D personality, compared with non-Type D personality, were not significantly different in age, body mass index, and the distribution of sex, NYHA functional classification, smoking, comorbidities, and use of medications, but the mean score of Beck Depression Inventory was significantly higher in patients with Type D personality (*P* < 0.001). A total of 55 patients (24.8%) and 89 patients (40.1%) had cardiac readmissions within 6 months and 18 months, respectively.

**Table 1:** Basic characteristics of patients with heart failure at baseline (*N* = 222).

Variable	n (%)			*P*
	Total	Type D	Non-Type D	
	222 (100)	23 (10.4)	199 (89.6)	
Sex, n (%)				0.410
female	75 (33.8)	6 (26.1)	69 (34.7)	
male	147 (66.2)	17 (73.9)	130 (65.3)	
Age, mean (SD), years	60.4 (12.1)	59.6 (11.8)	60.5 (12.2)	0.727
BMI, mean (SD), kg/m^2^ ^a^	26.0 (4.8)	25.7 (4.4)	26.0 (4.9)	0.784
NYHA functional classification, n (%)				0.844
II	61 (27.5)	6 (26.1)	55 (27.6)	
III	75 (33.8)	9 (39.1)	66 (33.2)	
IV	86 (38.7)	8 (34.8)	78 (39.2)	
Left ventricular ejection fraction (%)	42.8 (17.9)	41.3 (16.1)	43.0 (18.1)	0.659
Smoking, n (%)	99 (44.6)	11 (47.8)	88 (44.2)	0.742
BDI, mean (SD)	5.32 (6.01)	14.26 (9.75)	4.29 (4.41)	<0.001
Type 2 diabetes, n (%)	91 (41.0)	7 (30.4)	84 (42.2)	0.277
Kidney disease, n (%)	55 (24.8)	4 (17.4)	51 (25.6)	0.386
Hypertension, n (%)	139 (62.6)	12 (52.2)	127 (63.8)	0.274
Coronary heart disease, n (%)	55 (24.8)	7 (30.4)	48 (24.1)	0.507
Arrhythmia, n (%)	53 (23.9)	8 (34.8)	45 (22.6)	0.195
Medications, n (%)				
Diuretics	164 (73.9)	18 (78.3)	146 (73.4)	0.613
Beta-blockers	111 (50.0)	8 (34.8)	103 (51.8)	0.123
ACEIs	41 (18.5)	6 (26.1)	35 (17.6)	0.392
ARBs	89 (40.1)	7 (30.4)	82 (41.2)	0.318
Negative affectivity score, mean (SD)	6.53 (5.12)	15.43 (3.49)	5.50 (4.19)	<0.001
Social inhibition score, mean (SD)	6.05 (5.65)	15.00 (4.44)	5.02 (4.80)	<0.001
Negative affectivity score ≥ 10, n (%)	52 (23.4)	23 (100)	29 (14.6)	<0.001
Social inhibition score ≥ 10, n (%)	46 (20.7)	23 (100)	23 (11.6)	<0.001
6-month cardiac readmission, n (%)	55 (24.8)	10 (43.5)	45 (22.6)	0.028
18-month cardiac readmission, n (%)	89 (40.1)	13 (56.5)	76 (38.2)	0.089

ACEIs: Angiotensin-converting enzyme inhibitors; ARB: Angiotensin II receptor blockers; BMI: body mass index; BDI: Beck Depression Inventory; NYHA: New York Heart Association; SD: standard deviation.

^a^BMI has 6 missing values.

Table 2 shows the results of the univariate logistic regression analyses of Z-score transformed NA and SI, as well as the dichotomized NA and SI scores, for 6-month and 18-month cardiac readmission in patients with heart failure. For the 6-month cardiac readmission, the risk of readmission was significantly higher in patients with a higher Z-score transformed NA (odds ratio [OR] = 1.52 for a one SD increase in Z-score transformed NA, 95% confidence interval [CI] = 1.12–2.06, *P* = 0.007). The risk of readmission showed a trend of increase for patients with a higher Z-score transformed SI (OR = 1.34 for a one SD increase in Z-score transformed SI, 95% CI = 1.00–1.79, *P* = 0.052). Similar results were observed for the 18-month cardiac readmission. The risk of readmission was significantly higher in patients with a higher Z-score transformed NA (OR = 1.45 for a one SD increase in Z-score transformed NA, 95% CI = 1.10–1.92, *P* = 0.009). The risk of readmission showed a trend of increase for patients with a higher Z-score transformed SI (OR = 1.26 for a one SD increase in Z-score transformed SI, 95% CI = 0.96–1.65, *P* = 0.089). Furthermore, for the dichotomized scores, the risk of 6-month readmission was significantly higher in patients with either NA or SI (OR = 2.14, 95% CI = 1.09–4.20, *P* = 0.027) and OR = 2.13, 95% CI = 1.06–4.28, *P* = 0.034, respectively). The risk of 18-month readmission was significantly higher in patients with NA but marginally with SI (OR = 2.08, 95% CI = 1.11–3.91, *P* = 0.022) and OR = 1.86, 95% CI = 0.97–3.58, *P* = 0.062, respectively).

**Table 2:** Univariate logistic regression analyses of negative affectivity and social inhibition for 6-month and 18-month cardiac readmission in patients with heart failure (*N* = 222).

Variable	mean (SD) or *n* (%)			Odds ratio (95% CI)	*P*
	**6-month cardiac readmission**				
	**cardiac readmission**		**no cardiac readmission**		
	*n* = 55 (24.8%)		*n* = 167 (75.2%)		
Z(NA)	0.318 (1.070)		−0.108 (0.946)	1.52 (1.12–2.06)	0.007
Z(SI)	0.233 (0.940)		−0.072 (1.014)	1.34 (1.00–1.79)	0.052
NA score					
< 10	36 (65.5%)		134 (80.2%)	1.00	
≥ 10	19 (34.5%)		33 (19.8%)	2.14 (1.09–4.20)	0.027
SI score					
< 10	38 (69.1%)		138 (82.6%)	1.00	
≥ 10	17 (30.9%)		29 (17.4%)	2.13 (1.06–4.28)	0.034
	**18-month cardiac readmission**				
	**cardiac readmission**	**no cardiac readmission**			
	*n* = 89 (40.1%)	*n* = 133 (59.9%)			
Z(NA)	0.215 (1.037)	−0.148 (0.939)		1.45 (1.10–1.92)	0.009
Z(SI)	0.144 (0.936)	−0.091 (1.038)		1.26 (0.96–1.65)	0.089
NA score					
< 10 ≥ 10	61 (68.5%) 28 (31.5%)	109 (82.0%) 24 (18.0%)		1.00 2.08 (1.11–3.91)	0.022
SI score					
< 10 ≥ 10	65 (73.0%) 24 (27.0%)	111 (83.5%) 22 (16.5%)		1.00 1.86 (0.97–3.58)	0.062

SD: standard deviation. NA: negative affectivity. SI: social inhibition. Z(NA): Z-score transformed negative affectivity score. Z(SI): Z-score transformed social inhibition score

Table 3 shows the results of multiple logistic regression analyses of Z-score transformed NA and SI, as well as the dichotomized NA and SI scores, for 6-month and 18-month cardiac readmission in patients with heart failure. Both Z-score transformed NA and SI were significantly associated with either 6-month or 18-month cardiac readmission. The adjusted ORs of 6-month cardiac readmission were 1.62 (95% CI = 1.18–2.23, *P* = 0.003) and 1.48 (95% CI = 1.08–2.02, *P* = 0.014) for Z-score transformed NA and SI, respectively. The adjusted ORs of 18-month cardiac readmission were 1.45 (95% CI = 1.08–1.93, *P* = 0.013) and 1.38 (95% CI = 1.03–1.85, *P* = 0.031) for Z-score transformed NA and SI, respectively. Furthermore, for the dichotomized scores, the risk of 6-month readmission was significantly higher in patients with either NA or SI (adjusted OR = 2.08, 95% CI = 1.01–4.31, *P* = 0.048) and adjusted OR = 2.15, 95% CI = 1.03–4.47, *P* = 0.041, respectively). The risk of 18-month readmission was significantly higher in patients with NA but marginally with SI (adjusted OR = 2.00, 95% CI = 1.04–3.85, *P* = 0.038) and adjusted OR = 1.97, 95% CI = 0.98–3.94, *P* = 0.056, respectively).

**Table 3:** Multiple logistic regression analyses of negative affectivity and social inhibition for 6-month and 18-month cardiac readmission in patients with heart failure (*N* = 222).

Variable	Adjusted odds ratio (95% CI)	*P*
	**6-month cardiac readmission**	
Z(NA)^a^	1.62 (1.18–2.23)	0.003
Z(SI)^b^	1.48 (1.08–2.02)	0.014
NA score (≥ 10 vs < 10)^c^	2.08 (1.01–4.31)	0.048
SI score (≥ 10 vs < 10)^a^	2.15 (1.03–4.47)	0.041
	**18-month cardiac readmission**	
Z(NA)^d^	1.45 (1.08–1.93)	0.013
Z(SI)^e^	1.38 (1.03–1.85)	0.031
NA score (≥ 10 vs < 10)^d^	2.00 (1.04–3.85)	0.038
SI score (≥ 10 vs < 10)^e^	1.97 (0.98–3.94)	0.056

NA: negative affectivity. SI: social inhibition. Z(NA): Z-score transformed negative affectivity score. Z(SI): Z-score transformed social inhibition score. Potential confounding variables evaluated during model development included: sex, age, body mass index, smoking, left ventricular ejection fraction, New York Heart Association functional classification, Beck Depression Inventory, type 2 diabetes, kidney disease, hypertension, coronary heart disease, arrhythmia, diuretics, beta-blockers, angiotensin-converting enzyme inhibitors, and angiotensin II receptor blockers.

^a^Variables included in the adjusted model: sex, diuretics, and beta-blockers.

^b^Variables included in the adjusted model: diuretics and beta-blockers.

^c^Variables included in the adjusted model: sex, New York Heart Association functional classification, type 2 diabetes, hypertension, diuretics, and beta-blockers.

^d^Variables included in the adjusted model: coronary heart disease, diuretics, and angiotensin-converting enzyme inhibitors.

^e^Variables included in the adjusted model: coronary heart disease, diuretics, beta-blockers, and angiotensin-converting enzyme inhibitors.

## Discussion

Predicting readmission risk at the time of initial hospitalization in patients with heart failure can possibly provide better risk stratification, which allows for appropriate allocation of resources and targeted intervention. The present prospective study found that both Z-score transformed NA and SI were significantly associated with an increased risk of 6-month and 18-month cardiac readmission. The risk ranged from 1.38 for 18-month cardiac readmission in Z-score transformed SI to 1.62 for 6-month cardiac readmission in Z-score transformed NA, adjusting for other potential confounders. Similar findings were obtained when NA and SI were analyzed as dichotomized scores, except SI was only marginally (*P* = 0.056) associated with 18-month cardiac readmission.

NA has previously been shown to be positively and strongly correlated with neuroticism of the Big Five personality traits, whereas SI was inversely and strongly correlated with extraversion. Moderate correlations were observed for extraversion with NA, neuroticism with SI, and conscientiousness and agreeableness for both NA and SI [23, 24]. In patients with acute coronary syndrome, a personality type characterized by higher neuroticism and lower extraversion, agreeableness, and conscientiousness was associated with a poorer outcome of depression [25]. Moreover, a systematic review and meta-analysis of prospective cohort studies indicated that anger and hostility were significantly associated with poor prognosis in patients with existing coronary heart disease. The association might be mediated through poor health behavior, such as poor diet, less physical activity, smoking, poor sleep, and lower treatment adherence [26]. These results are consistent with the findings in the present study that psychological factors, such as NA and SI, can play a role in the clinical outcome of patients with heart failure.

A number of potential pathophysiological pathways could be involved in the association of NA and SI on the increased risk of cardiac readmission. First, imbalance in the autonomic nervous system may play a role in the adverse cardiac events. A recent study in patients with coronary artery disease showed that NA was inversely correlated with standard deviation of all normal-to-normal intervals (SDNN) and total power of heart rate variability (HRV) [27]. In patients with heart failure, Type D personality was associated with an inadequate heart rate response [28], and there is strong evidence linking autonomic imbalance with heart failure [29].

Second, inflammation can play a pivotal role in the pathophysiology of heart failure [30]. A study on 228 patients with heart failure reported that Type D personality and change in self-reported physical health status over 18 months was significantly mediated by a number of inflammatory biomarkers [31]. Type D personality was shown to be associated with increased tumor necrosis factor-alpha (TNF-alpha) levels, which have been implicated in the pathogenesis of heart failure [32]. In addition, patients with Type D personality had significantly higher levels of the inflammation marker high-sensitivity C-reactive protein (hsCRP) compared with those with non-Type D personality [33], and a higher hsCRP was found to be an independent predictor of higher rates of readmission in patients with heart failure [34].

Third, endothelial function is a critical component in the systemic vasodilation in patients with heart failure [35]. A study on patients with heart failure found a significantly lower level of circulating CD34+/ kinase insert domain-containing receptor (KDR) + endothelial progenitor cells in patients with Type D personality, which might explain the association between impaired endothelial function in patients with Type D personality and heart failure [36]. In addition, a recent prospective study on 180 patients with coronary artery disease reported that Type D personality could predict impaired endothelial function in men [37].

The results of this study should be interpreted in light of its limitations. First, the role of NA and SI components of Type D personality might vary for different types of heart failure, including right and left sided dysfunction, in isolation or together. Nevertheless, our sample size was not sufficient to allow for more refined stratification of the type of heart failure. Second, the study subjects were recruited from only two regional hospitals and may limit the generalizability of study findings. Third, the number of patients with Type D personality was only 23 and therefore, our findings should be considered as preliminary. Further studies involving a larger number of samples are needed to confirm our results. Fourth, while the mean score of the Beck Depression Inventory was observed to be significantly higher in patients with Type D personality in the present study and previous research has indicated that depression could exacerbate coexisting chronic heart failure and its clinical outcomes [38], severity of depression in our study participants did not appear to affect our findings. Nevertheless, the possible influence of other unmeasured variables, such as social support [39] on our findings was not evaluated and thus, the presence of potential confounding effects could not be completely ruled out.

In conclusion, the present prospective study in patients with heart failure suggested that NA and SI components of the Type D personality were significantly associated with a higher risk of cardiac readmission in both 6 months and 18 months after the initial hospitalization. If the above relationship could be reliably demonstrated in replication studies, the role for psychiatric treatment on cardiovascular outcomes should be considered and evaluated.

## Supporting information

S1 Dataset Dataset of the study. (SAV)
